# Expression of Senescence and Osteoporosis-Associated microRNAs in Human Bone and Their Correlations With Clinical Parameters of Osteoporotic Patients

**DOI:** 10.33549/physiolres.935778

**Published:** 2026-06-01

**Authors:** Kveta VRABLIKOVA, Petr HOZA, Helena PAROVA, Marian RYBAR, Japneet KAUR, Sundeep KHOSLA, David MONROE, Petra SAUER MYKISKOVA, Vladimir PALICKA

**Affiliations:** 1 Department of Clinical Biochemistry, University Hospital Hradec Kralove, Faculty of Medicine Charles University in Hradec Kralove, Czech Republic; 2 Orthopaedic Department, Pardubice Hospital, Czech Republic; 3 Department of Biomedical Technology, Faculty of Biomedical Engineering, Czech Technical University in Prague, Kladno, Czech Republic; 4 University of Arkansas for Medical Sciences, Little Rock, AR, USA; 5 Division of Endocrinology, Department of Medicine, Mayo Clinic College of Medicine, Rochester, MN, USA; 6 Robert and Arlene Kogod Center on Aging, Mayo Clinic, Rochester, MN, USA

**Keywords:** Cellular senescence, Bone metabolism, Osteoporosis, Bone biopsies, miRNA

## Abstract

Osteoporosis is an age-related disease associated with impaired bone remodeling and cellular senescence. MicroRNAs (miRNAs) play an important regulatory role in these processes. This study aimed to identify senescence-associated miRNAs in human osteoporotic bone tissue and to assess their relationship with clinical parameters. A total of 46 patients undergoing total hip arthroplasty were included in this single-center cross-sectional study, representing a relatively large and unique cohort of human bone tissue samples: 21 with osteoporotic proximal femur fracture and 25 with elective arthroplasty for osteoarthritis as controls. Total RNA was isolated from femoral neck biopsies, and the relative expression of 11 selected miRNAs was quantified by qRT-PCR and expressed using 2^^−ΔΔCt^ method. Data were analyzed using *t*-tests, correlation analysis, and generalized linear models (GLM). MiR-106a-5p was significantly upregulated in osteoporotic patients, whereas miR-34a-5p, miR-218-5p, and miR-155-5p were significantly downregulated. After adjustment for clinical variables, miR-106a-5p remained independently positively associated with osteoporosis, while miR-34a-5p and miR-218-5p showed independent negative associations. MiR-92a-3p and miR-181a-5p became significantly associated with osteoporosis only after adjustment. Network analysis linked the dysregulated miRNAs to genes involved in DNA damage response and cell-cycle control. Osteoporosis is associated with a specific senescence-related miRNA signature in human bone tissue. Integration of molecular and clinical data improved the interpretation of miRNA-disease associations and supports the potential of selected miRNAs as biomarkers of bone aging and osteoporosis.

## Introduction

In the context of a globally aging population, there is growing interest in understanding the pathophysiological mechanisms underlying chronic diseases prevalent in older age [1]. The rising incidence of age-related conditions such as osteoporosis and low-trauma fractures associated with reduced bone density highlights the critical need to address their underlying causes. It is estimated that worldwide, one in three women and one in five men over the age of 50 will experience an osteoporotic fracture during their remaining lifetime [2,3]. Data from the United States indicate that hospitalization-related costs are highest for osteoporotic fractures ($5.1 billion), followed by myocardial infarction ($4.3 billion), stroke ($3.0 billion), and breast cancer ($0.5 billion) [4]. Age-associated bone diseases, including osteoporosis, are a major contributor to reduced quality of life in the elderly and often result in prolonged hospitalization, disability, and loss of independence [3,5,6].

Currently, the recommended tool for diagnosing and monitoring osteoporosis and assessing fracture risk is the measurement of bone mineral density (BMD) using dual-energy X-ray absorptiometry (DXA) [7]. However, BMD has limited sensitivity and reflects only part of overall fracture risk [3,8]. Patients with a recent fracture are at a particularly high risk of sustaining subsequent fractures, largely independent of their baseline T-score [9].

Aging at the cellular level is characterized by several hallmarks, including cellular senescence, which is driven by increased activity of cyclin-dependent kinase inhibitors, particularly *CDKN1A* (p21^Cip1^) and *CDKN2A* (p16^INK4a^) [10]. Senescent cells are defined by persistent cell-cycle arrest accompanied by morphological and functional changes, sustained metabolic activity, and increased resistance to apoptosis mediated by senescence-associated anti-apoptotic pathways [11]. Regulatory mechanisms, inducers, and biomarkers of cellular senescence include telomere dysfunction, DNA damage, chromatin remodeling, dysregulation of reactive oxygen species, accumulation of mitochondria and lysosomes, and altered miRNA expression. Senescent cells also exhibit a senescence-associated secretory phenotype (SASP), characterized by the production of chemokines, pro-inflammatory cytokines, and matrix-degrading proteins [12–14]. The accumulation of senescent cells and SASP contributes to aging processes in multiple tissues, including bone [8,9,10]. Identification of senescence-inducing pathways in bone has enabled the search for biomarkers and therapeutic targets with the aim of extending health span [6]. There is a growing rationale for therapeutic strategies targeting senescent cells or suppressing SASP activity, including the use of senolytics [11].

Genetic modification represents another approach, with currently available genome-editing tools including zinc finger nucleases (ZFNs) and transcription activator-like effector nucleases (TALENs) [7,15,16]. The most recent breakthrough, clustered regularly interspaced short palindromic repeats/CRISPR-associated protein 9 (CRISPR-Cas9), has led to the first CRISPR-based gene therapies approved by the FDA and EMA for monogenic diseases [15–19]. For any targeted therapeutic intervention, it is essential to understand the regulatory mechanisms, inducers, and biomarkers of bone senescence, including miRNA-mediated regulation [11].

MicroRNAs are small non-coding ribonucleic acids (~22 nucleotides in length) that post-transcriptionally regulate gene expression [20]. Partial complementarity between a miRNA and its target messenger RNA (mRNA) results in destabilization and/or translational repression, while near-perfect complementarity leads to direct cleavage of the target mRNA [21]. Further research is needed to elucidate these complex regulatory processes at both cellular and extracellular levels [22]. Understanding the role of miRNAs in human disease is essential for uncovering disease mechanisms and developing novel therapeutic approaches [23–25]. The importance of this field was recently highlighted by the awarding of the 2024 Nobel Prize in Physiology or Medicine to Victor Ambros and Gary Ruvkun for their discovery of microRNAs [26].

One of the major challenges in miRNA biology remains the complexity of miRNA-mRNA interactions, where a single miRNA can regulate multiple mRNAs and each mRNA can be targeted by several miRNAs [27]. We hypothesized that specific miRNAs are differentially expressed in senescent human bone tissue. The aim of this study was to identify such miRNAs in human femoral neck bone samples and to evaluate their association with baseline clinical characteristics in patients undergoing total hip arthroplasty in a real-world setting.

## Materials and Methods

### Ethical approval

Presented study was approved by the Ethics Committee of University Hospital Hradec Kralove (201905 S10P).

### Senescence, osteoporosis-associated miRNA panel

**Fig. 1:**
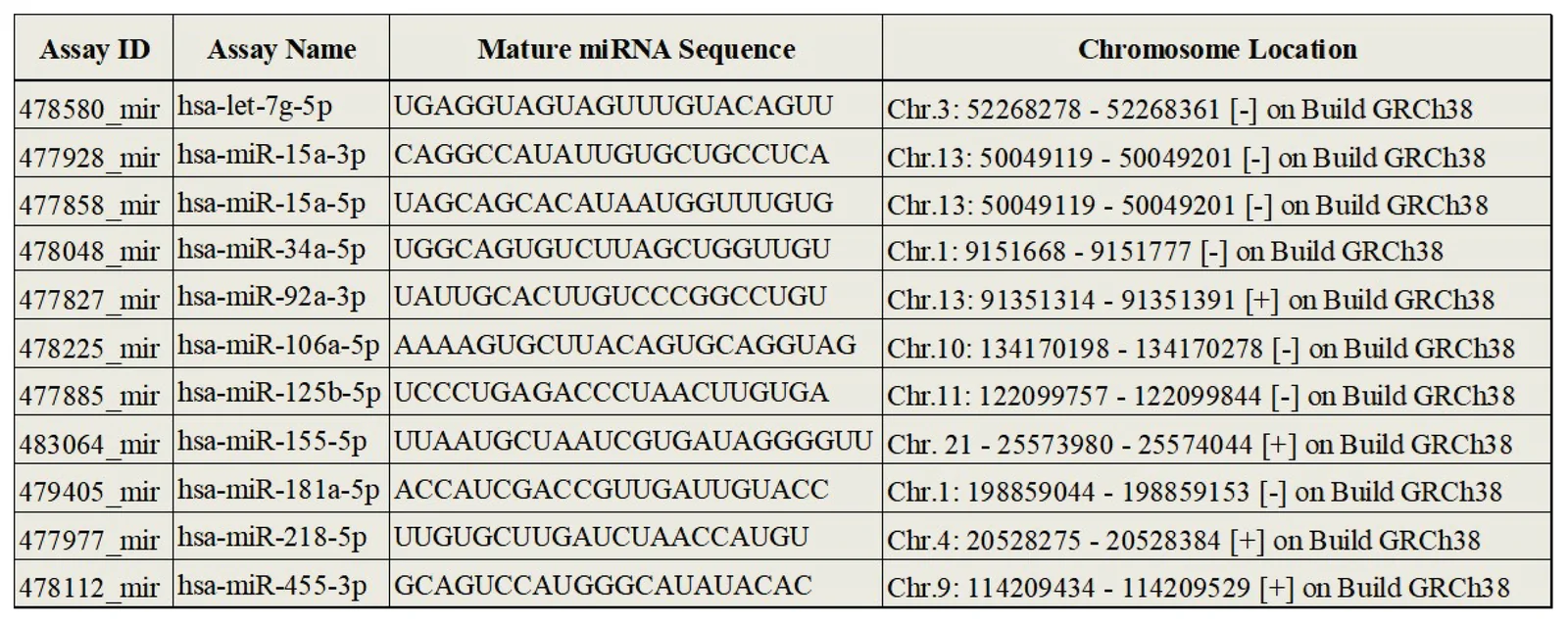
The panel of identified miRNA (miRNet database).

The miRNA panel was selected to capture the overlap between senescence- and osteoporosis-associated miRNAs. Based on data from the miRNet database and a literature review of human studies, the following miRNAs were identified: hsa-miR-7g-5p, hsa-miR-15a-3p, hsa-miR-15a-5p, hsa-miR-34a-5p, hsa-miR-92a-3p, hsa-miR-106a-5p, hsa-miR-125b-5p, hsa-miR-155-5p, hsa-miR-181a-5p, hsa-miR-218-5p, and hsa-miR-455-3p (Fig. 1), hsa-miR-361-5p and hsa-miR-16-5p were used as endogenous controls.

### Study design

A single-center cross-sectional observational study was approved by the Ethics Committee of University Hospital Hradec Kralove (No. 201905 S10P). The approved protocol included the collection of fresh femoral neck bone biopsies that would otherwise be discarded during orthopedic surgery, as well as peripheral blood samples. Surgery in patients with fracture was performed within 24–48 h according to standard orthopedic department procedures. Bone biopsies were obtained from patients undergoing total hip arthroplasty either for low-trauma proximal femur fracture or for elective indications due to osteoarthritis. All samples were collected during endoprosthesis implantation without any additional burden to the patients. Written informed consent was obtained from all participants prior to sample collection.

### Study subjects

Participants (n=46) were enrolled at the Department of Orthopaedics, Pardubice Hospital, between 2020 and 2022. The study included postmenopausal women and men aged 49–88 years (mean ± SD: 70.4±7.8 years) who underwent total hip arthroplasty. Patients were stratified into two groups according to the indication for surgery:

1. patients with a low-trauma osteoporotic proximal femur fracture (OP Group; n=21); and
2. patients undergoing elective total hip arthroplasty for primary osteoarthritis without a prior osteoporotic fracture (Control Group; n=25).

The two groups were compared under the null hypothesis that there is no difference in the expression of aging- and osteoporosis-associated miRNAs between the groups. Study screening included medical history assessment, dual-energy X-ray absorptiometry (DXA), and blood sampling for biochemical analyses, including C-terminal telopeptide of type I collagen (CTX-I) and procollagen type I N-terminal propeptide (P1NP).

### Collection of bone samples

Femoral neck bone biopsies were obtained from patients undergoing total hip arthroplasty either for low-trauma proximal femur fracture (n=21) or for elective indications due to osteoarthritis (n=25). All procedures were performed using the anterolateral Bauer approach. Bioptic bone samples were collected from the excised femoral neck following its removal during standard total hip arthroplasty, in accordance with approved study protocols; the procedure did not require any additional surgical intervention.

A cylindrical bone specimen was obtained from the central part of the femoral neck using a biopsy trephine with an internal diameter of 3 mm. Bone tissue from the immediate osteotomy margin was avoided to minimize the risk of mechanical or thermal damage. The collected specimen (5–8 mm in length) was immediately transferred into 1.5 ml of TRIzol™ reagent (Invitrogen, Gaithersburg, MD, USA) and stored at −80 °C until RNA isolation.

### Homogenization of bone samples and RNA isolation

Bone samples were transferred into homogenization tubes (SeptiFast Lys Kit, Roche Diagnostics, Basel, Switzerland) containing 850 μl of QIAzol Lysis Reagent (Qiagen, Hilden, Germany) and homogenized using the MagNA Lyser (Roche Diagnostics) for 90 s at 7,000× g. When required, an additional 30 s of homogenization was performed. The tubes were immediately placed on a frozen rack after homogenization.

Following centrifugation, 700 μl of the homogenate was transferred to a new 1.5 ml tube and incubated for 5 min at room temperature. Total RNA, including miRNA, was isolated using the miRNeasy Mini Kit (Qiagen) according to the manufacturer’s protocol with minor modifications. Chloroform (140 μl) was added, the samples were mixed vigorously for 15 s, incubated for 2–3 min at room temperature, and centrifuged for 15 min at 12,000× g at 4 °C.

The upper aqueous phase was carefully transferred to a new tube, mixed with 1.5 volumes of 96 % ethanol, and loaded onto the spin column in two consecutive steps (700 μl each), followed by centrifugation for 15 s at 8,000× g. The column was washed with 700 μl of RWT buffer and 500 μl of RPE buffer, each followed by centrifugation at 8,000× g for 15 s.

RNA was eluted in 30 μl of RNase-free water. RNA concentration and purity were determined using a NanoDrop ND-1000 spectrophotometer (Thermo Fisher Scientific, Wilmington, DE, USA) by measuring absorbance at 260 and 280 nm (A_260_/_280_). Isolated RNA was immediately processed or stored at −80 °C.

### Quantitative Real-time PCR of miRNAs

cDNA was synthesized using the TaqMan® Advanced miRNA cDNA Synthesis Kit with universal reverse transcription primers (Applied Biosystems, Foster City, CA, USA) according to the manufacturer’s protocol, using 8–10 ng of total RNA per reaction. Quantitative real-time PCR was performed using TaqMan® Fast Advanced Master Mix and specific TaqMan® Advanced miRNA Assays (Applied Biosystems, USA) on the Rotor-Gene Q system (Qiagen).

Assays for hsa-let-7g-5p, hsa-miR-15a-3p, hsa-miR-15a-5p, hsa-miR-34a-5p, hsa-miR-92a-3p, hsa-miR-106a-5p, hsa-miR-125b-5p, hsa-miR-155-5p, hsa-miR-181a-5p, hsa-miR-218-5p, and hsa-miR-455-3p were used (Fig. 1). Hsa-miR-361-5p and hsa-miR-16-5p served as endogenous controls. All reactions were performed in triplicate in a final volume of 10 μl, with 2.5 μl of cDNA per reaction.

Thermal cycling conditions were as follows: enzyme activation at 95 °C for 20 s, followed by 40 cycles of denaturation at 95 °C for 3 s and annealing/extension at 60 °C for 30 s. Fluorescence data were analyzed using Rotor-Gene Q Series Software. Relative miRNA expression was calculated using the 2^^−ΔΔCt^ method, with normalization to hsa-miR-361-5p and hsa-miR-16-5p. The workflow and endogenous controls were selected based on both published literature and manufacturer recommendations [28].

### Statistical analyses

Baseline characteristics are presented as mean ± standard deviation (SD). Differences between the osteoporosis (OP) and control groups were analyzed using the two-sided Student’s *t*-test for independent samples, with p<0.05 considered statistically significant. Normality of data distribution was verified using the Shapiro-Wilk test.

Ct values obtained by qRT-PCR in triplicate were averaged for each sample. Group comparisons were performed using ΔCt values, where lower ΔCt indicates higher miRNA expression. Relative expression was calculated as fold change (FC) using the 2^^−ΔΔCt^ method with selected endogenous controls. Significant upregulation was defined as p<0.05 and FC>2.0, and significant downregulation as p<0.05 and FC<0.5. FC values were log_2_-transformed and visualized using volcano plots.

Associations between miRNA expression and clinical parameters were analyzed using univariate correlations, followed by multivariate generalized linear models (GLM) for variables with p<0.25. Selected baseline characteristics of the study population (femoral neck BMD, BMI, CTX-I, P1NP, and age) were included in a correlation matrix for univariate analysis. Variables with a p<0.25 were subsequently entered into multivariate GLM to identify independent associations between the expression of individual miRNAs (dependent variable) and baseline characteristics (independent variables). This more liberal p-value threshold (0.25) is intentionally used to avoid premature exclusion of variables with potential confounding or independent effects that may become evident after multivariable adjustment. Age and group affiliation (OP vs. control) were included as mandatory covariates in all models.

MiRNA expression was considered the dependent variable, while clinical parameters were included as independent variables; age and group affiliation (OP vs. control) were mandatory covariates in all models. Data were analyzed using R Commander, with statistical significance set at p<0.05.

## Results

### Baseline examination

The basic demographic and clinical characteristics of the study population are summarized in Table 1. The mean age was 72±7.29 years in the osteoporosis (OP) group and 69±8.06 years in the control group (p=0.29). The proportion of women was comparable between groups, with 67 % (n=14) in the OP group and 72 % (n=18) in the control group (p=0.70). The proportion of men was also similar, with 33 % (n=7) in the OP group and 28 % (n=7) in the control group (p=1.00). Smoking prevalence did not differ between groups, being 40 % (n=10) in the OP group and 43 % (n=9) in the control group (p=0.85).

Body mass index (BMI) was significantly lower in the OP group compared with controls (27±3.43 vs. 30±4.65 kg/m^2^; p=0.01), whereas femoral neck BMD did not differ between groups (1.0±0.18 vs. 1.0±0.20 g/cm^3^; p=0.56).

Serum CTX-I levels were significantly higher in the OP group than in controls (938.5±646.8 vs. 594.0±285.3 ng/l; p=0.04). According to the reference range used at Pardubice Hospital for adults aged 18–99 years (70–700 ng/l), the mean CTX-I level in the OP group exceeded the upper reference limit, while control values were within the normal range.

Serum P1NP levels were higher in the OP group than in controls (93.2±85.5 vs. 59.5±24.4 μg/l), although this difference did not reach statistical significance (p=0.07). The reference range for P1NP at Pardubice Hospital is 20–70 μg/l. The mean P1NP value in the OP group exceeded the upper reference limit, while control values were within the upper normal range.

Mean serum 25(OH)D concentrations were 39.4±20.2 nmol/l in the OP group and 44.1±20.6 nmol/l in the control group, with no statistically significant difference between the groups (p=0.47); both groups exhibited values below the reference range (75–250 nmol/l). Laboratory analysis revealed a marked vitamin D deficiency in both groups.

### miRNA expression

**Fig. 2:**
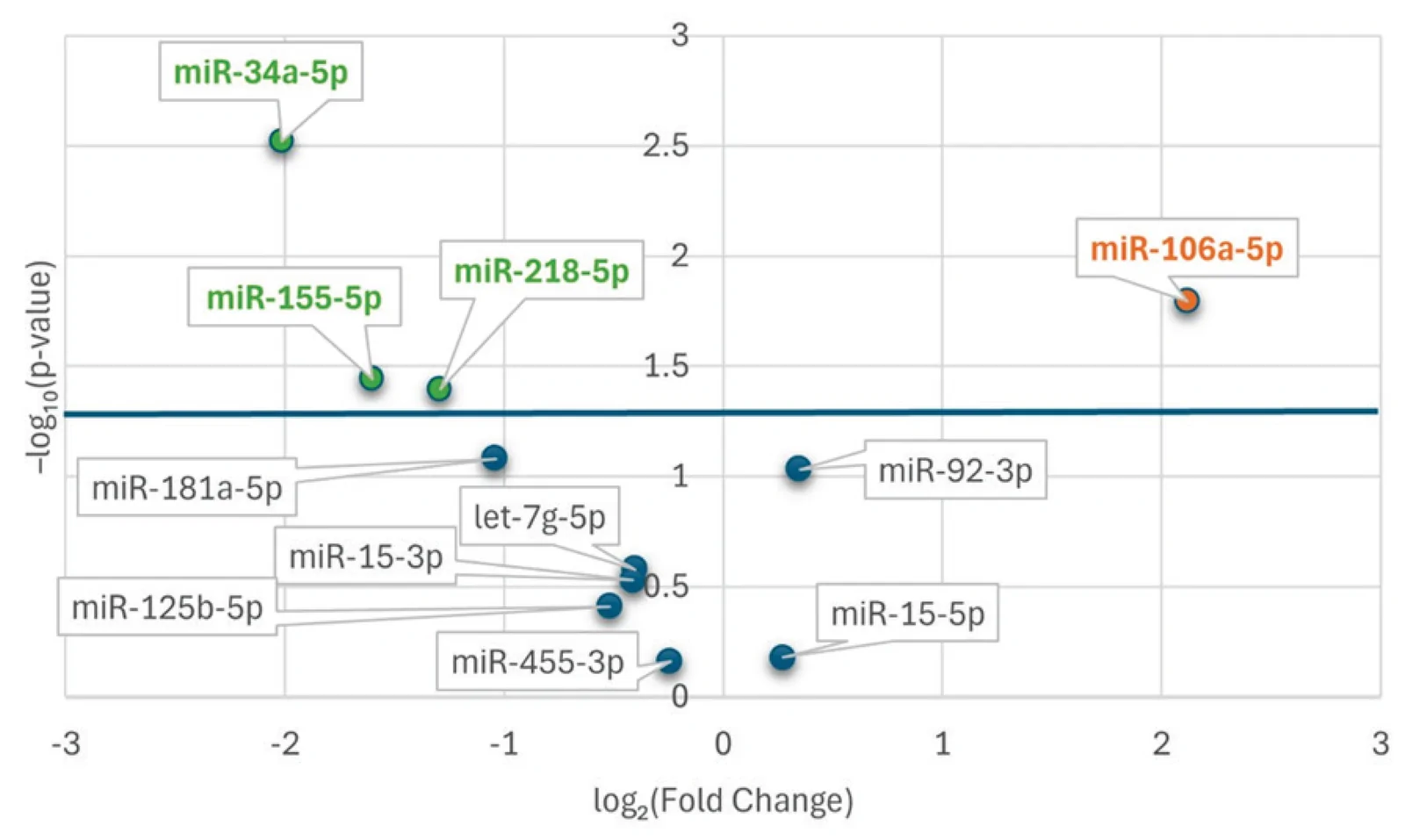
Volcano Plot of Logarithmic Changes in miRNA Expression between the OP and Control Groups. The X-axis displays log_2_(FC), and the Y-axis shows -log_10_(p-value). The horizontal blue line marks the threshold for statistical significance (p<0.05). Vertical lines indicate the cut-offs for biologically meaningful regulation (|log_2_FC|=1). Green-labeled miRNAs (left) are significantly downregulated, while the orange-labeled miRNA (right) is significantly upregulated.

Out of 46 involved patients, miRNA expression was reliably quantified in 35 of them (OP: n=16; control: n=19) and this group of patients was used for further analysis. Among the analyzed miRNAs, miR-106a-5p showed the strongest and the only statistically significant upregulation in the OP group (FC=4.34, p=0.016). MiR-92a-3p and miR-15a-5p exhibited a mild, but non-significant, tendency toward upregulation (FC=1.27, p=0.086 and FC=1.21, p=0.684, respectively). In contrast, miR-34a-5p was the most strongly downregulated miRNA (FC=0.25, p=0.004), followed by significant downregulation of miR-155-5p (FC=0.33, p=0.046) and miR-218-5p (FC=0.41, p=0.034), while miR-181a-5p showed a non-significant trend toward downregulation (FC=0.49, p=0.078). No significant differences were observed for let-7g-5p, miR-15a-3p, miR-125b-5p or miR-455-3p. For visualization of the magnitude and direction of expression changes, FC values were log_2_-transformed and displayed in a volcano plot (Fig. 2).

### Independent associations between microRNA expression and baseline characteristics of the study population

Selected baseline characteristics of the study population (femoral neck BMD, BMI, CTX-I, P1NP, and age) were included in a correlation matrix for univariate analysis. Variables with a p<0.25 were subsequently entered into multivariate GLM to identify independent associations between the expression of individual miRNAs (dependent variable) and baseline characteristics (independent variables). Age and group affiliation (OP vs. control) were included as mandatory covariates in all models.

After adjustment for the respective independent variables, no significant associations were observed for miR-15a-3p, miR-15a-5p, miR-125b-5p, miR-155-5p, or miR-455-3p.

Expression of miR-34a-5p, after adjustment for age and osteoporosis status, showed a statistically significant negative association with OP (p<0.05).

Expression of miR-92a-3p, after adjustment for femoral neck BMD, BMI, CTX-I, P1NP, age, and OP status, showed a statistically significant positive independent association with OP (p<0.05).

Expression of miR-106a-5p, after adjustment for CTX-I, P1NP, age, and OP status, also showed a statistically significant positive independent association with OP (p<0.05).

Expression of miR-181a-5p, after adjustment for BMI, CTX-I, age, and OP status, showed a statistically significant negative independent association with both OP and CTX-I (p<0.05).

Expression of miR-218-5p, after adjustment for BMI, CTX-I, age, and OP status, showed a statistically significant negative independent association with OP (p<0.05).

The individual models are presented in detail in Table 2.

## Discussion

### Baseline characteristics and bone turnover markers

Baseline demographic parameters were well balanced between groups. Age, sex distribution and smoking prevalence did not differ between the OP and control groups. BMI was significantly lower in the OP group compared with controls, consistent with the known association between low body weight and increased fracture risk.

Bone turnover markers, including CTX-I and P1NP, are known to increase with age, which partly explains their elevated levels in our cohort. However, despite comparable age characteristics, CTX-I levels were significantly higher in the OP group, with a similar trend observed for P1NP. These differences cannot be attributed to age alone and may, at least in part, reflect recent fracture-related activation of bone remodeling in addition to underlying osteoporotic bone metabolism [29]. Both groups exhibited marked vitamin D deficiency, with mean serum 25(OH)D levels well below the reference range, consistent with the high prevalence of hypovitaminosis D in older populations and representing an unfavorable factor for bone metabolism and fracture risk [30].

### Deregulated miRNA expression in osteoporosis

MiR-106a-5p showed the strongest and the only statistically significant upregulation in the OP group (FC≈4). MiR-92a-3p and miR-15a-5p exhibited a non-significant trend toward upregulation (FC slightly >1). In contrast, miR-34a-5p was strongly downregulated (FC≈0.25), while miR-155-5p, miR-181a-5p, and miR-218-5p showed moderate downregulation (FC≈0.33–0.49). Let-7g-5p, miR-15a-3p, miR-125b-5p and miR-455-3p showed no significant deregulation.

### Independent associations with clinical parameters (GLM)

In multivariate generalized linear models adjusted for clinical parameters identified by univariate correlation, miR-106a-5p and miR-92a-3p showed independent positive associations with osteoporosis. Although BMI was the only clinical parameter that differed significantly between the groups, these associations remained significant after adjustment for BMI. In contrast, miR-34a-5p, miR-181a-5p, and miR-218-5p showed independent negative associations with osteoporosis, with an additional inverse association between miR-181a-5p and CTX-I. No independent associations were observed for miR-15a-3p, miR-15a-5p, miR-125b-5p, miR-155-5p or miR-455-3p.

### Biological implications of dysregulated miRNAs

**Fig. 3:**
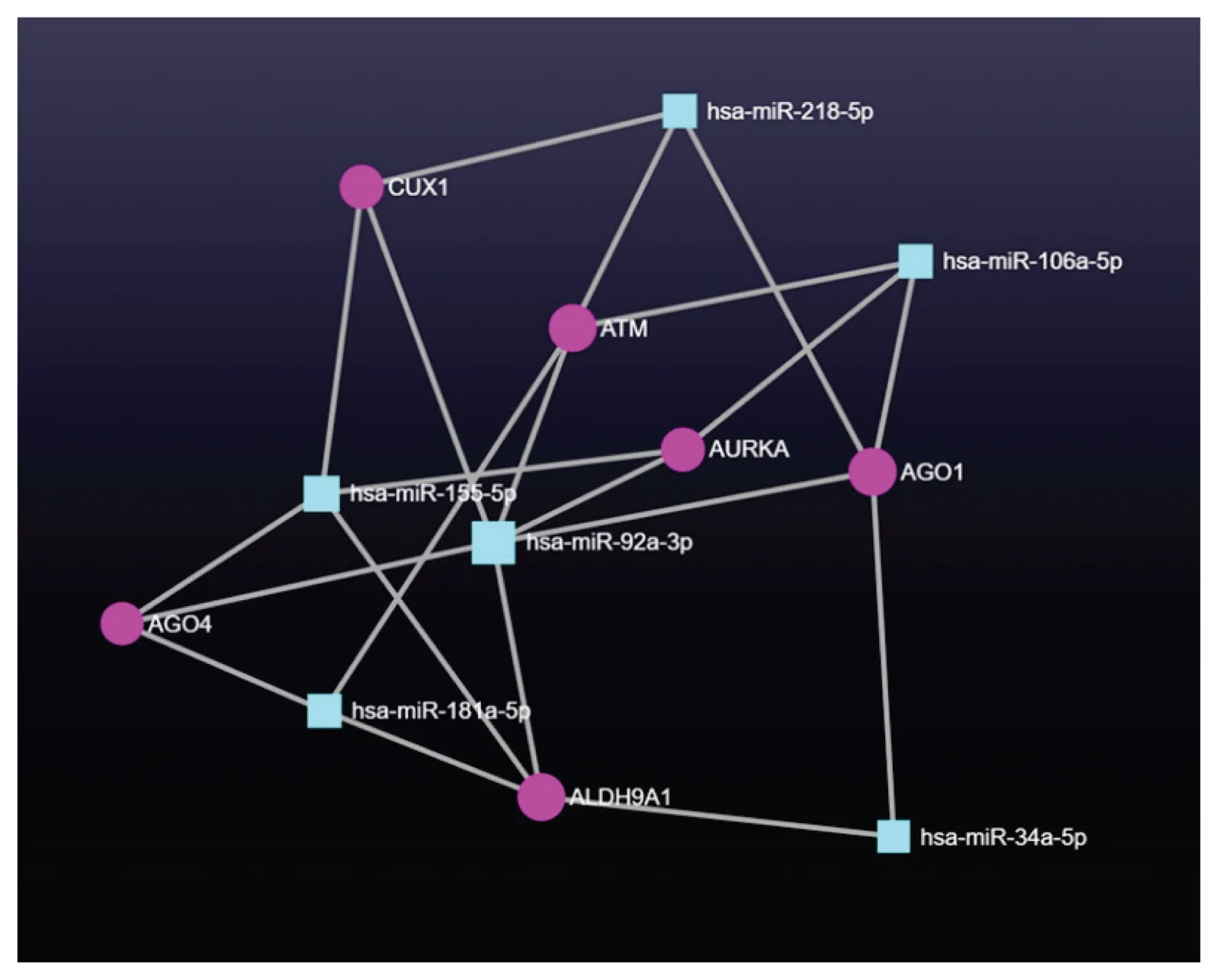
miRNA connections with shared target genes identified using miRNet.

The miRNet analysis showed that the studied miRNAs are connected to several shared target genes (Fig. 3). These genes form key regulatory nodes involved in DNA damage response, cell-cycle control and pathways linked to cellular senescence. This supports the idea that dysregulated miRNAs may contribute to impaired bone homeostasis through senescence-related mechanisms.

Notably, while miR-106a-5p was markedly upregulated in our study, previous experimental data indicate that oxidative stress leads to its downregulation. This apparent discrepancy suggests that increased miR-106a-5p expression in our cohort may represent a compensatory or context-dependent response to cellular stress, accelerated remodeling, or senescence-related ATM-dependent DNA damage signaling. Its predicted links to ATM, AURKA and AGO1 further suggest involvement in cell-cycle regulation and stress signaling [31,32].

miR-34a-5p showed the strongest downregulation. This miRNA normally suppresses osteoclast activity, and reduced levels may therefore contribute to disturbed bone remodeling. Its predicted connections to AGO1 and ALDH9A1 suggest additional roles in metabolic and stress-response pathways relevant to senescence [33].

miR-218-5p was significantly downregulated in the OP group, consistent with its known osteoanabolic role in promoting osteoblast differentiation and supporting Wnt signaling. Reduced miR-218-5p may therefore contribute to impaired osteogenesis and diminished bone formation in osteoporosis. miRNet analysis showed predicted links of miR-218-5p to ATM, CUX1 and AGO1, suggesting additional involvement in DNA damage response, transcriptional control and miRNA-mediated silencing. This network position indicates that its downregulation may also influence senescence-related pathways and increase cellular vulnerability to stress [34,35].

miR-92a-3p showed only a trend in univariate analysis, but after adjustment it was independently associated with osteoporosis. This is consistent with its known role in promoting osteoblast differentiation and bone repair *via* the IBSP/PI3K-AKT pathway. Its network links to ATM, AURKA and ALDH9A1 point to a possible interaction with senescence and metabolic control during fracture healing [36].

miR-181a-5p regulates osteogenic differentiation and was inversely associated with CTX-I. This supports its role in the control of bone resorption. Its predicted links to ALDH9A1, AGO4 and ATM suggest involvement in stress responses and metabolic stability of bone cells [37].

miR-155-5p was significantly downregulated. This miRNA participates in p53/p21 and NF-κB signaling and influences MSC differentiation toward adipogenesis. Its links to CUX1, AURKA, AGO4 and ALDH9A1 indicate a role in transcriptional control, cell-cycle regulation and senescence-related processes [38].

Let-7g-5p, miR-15a-3p, miR-125b-5p and miR-455-3p did not show significant changes [39,40]. These miRNAs are therefore unlikely to act as major biomarkers of osteoporosis in this cohort.

### Potential target genes of candidate miRNAs

**Fig. 4:**
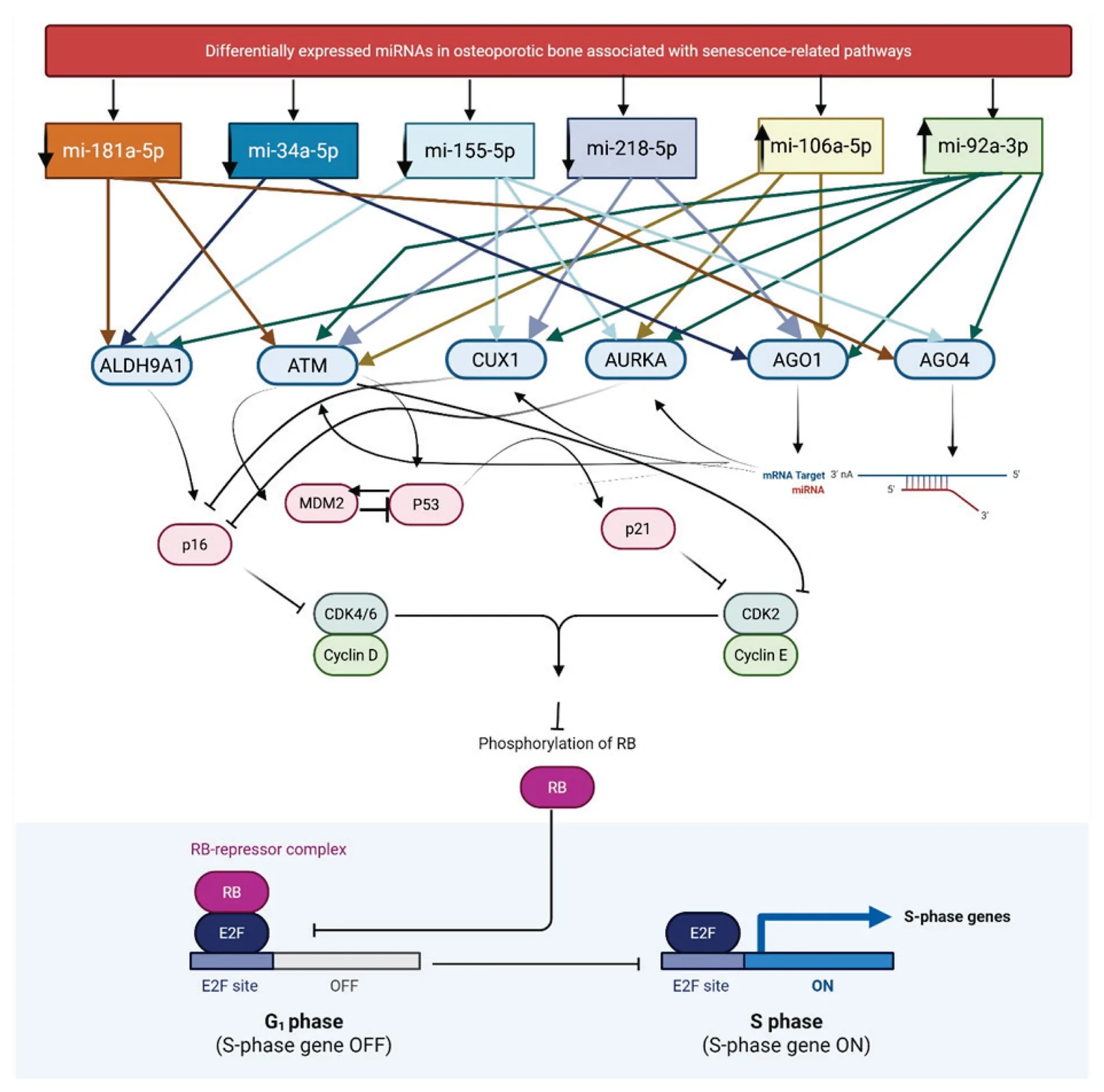
Potential target genes of candidate miRNAs (Created with BioRender.com).

Several target genes emerged as central nodes in the miRNA-gene interaction network (Fig. 4). AGO1 and AGO4 are components of the Argonaute family involved in miRNA-mediated post-transcriptional silencing. AGO1 participates in RISC activity and thereby influences cell-cycle control, stress responses and senescence. AGO4, although catalytically weaker, also contributes to miRNA regulation and affects epigenetic mechanisms such as DNA methylation and histone modification [41]. In our dataset, AGO1 was linked to miR-34a-5p, miR-92a-3p, miR-106a-5p and miR-218-5p, whereas AGO4 interacted with miR-92a-3p, miR-155-5p and miR-181a-5p. These patterns suggest that both proteins integrate regulatory signals related to senescence and bone metabolism, but each within distinct miRNA circuits.

ALDH9A1 is an enzyme involved in aldehyde and amino-acid metabolism and protects cells from oxidative stress [42]. It was identified as a predicted target of miR-34a-5p, miR-92a-3p, miR-155-5p and miR-181a-5p. This overlap indicates that altered miRNA expression may influence metabolic stability of bone cells and their ability to adapt to stress, contributing to senescent features in osteoporotic tissue.

AURKA functions as a negative regulator of senescence in mesenchymal stem cells. Its upregulation suppresses p16, p53 and p21, whereas AURKA depletion enhances senescence. In our network, AURKA was linked to miR-106a-5p, miR-155-5p and miR-92a-3p. These interactions suggest that changes in these miRNAs may modulate senescence pathways through AURKA-dependent mechanisms.

ATM is a key sensor of DNA double-strand breaks and activates the ATM-p53-p21 cascade, leading to cell-cycle arrest, DNA repair or senescence [43]. It was predicted as a target of miR-106a-5p, miR-218-5p, miR-92a-3p and miR-181a-5p. Each of these miRNAs is functionally linked to processes central to bone homeostasis–cell-cycle regulation, osteoblast differentiation, osteogenic signaling or bone resorption. Their combined dysregulation may therefore modify ATM activity and shift the balance between DNA repair and senescence in bone cells, with consequences for osteoblast function and bone remodeling [44].

CUX1 is a transcription factor involved in cell-cycle control, differentiation and stress responses. Recent studies identified CUX1 as a direct repressor of p16INK4a, indicating its essential role in maintaining proliferative capacity during aging [45]. In our network, CUX1 was linked to miR-92a-3p, miR-155-5p and miR-218-5p. These interactions suggest a potential regulatory axis through which altered miRNA expression may influence the p16-CDK4/6-RB pathway and contribute to the senescent phenotype observed in osteoporotic bone tissue.

### Study Limitations

The available data do not allow a clear distinction as to whether the observed changes in miRNA expression are primarily related to osteoporosis or reflect the presence of a recent low-trauma fracture. This limitation should be considered when interpreting the results and represents an area for further investigation.

## Conclusions

This study demonstrates that osteoporosis is associated with a senescence-related miRNA signature directly detectable in a relatively large cohort of human femoral neck bone samples. MiR-106a-5p was upregulated, whereas miR-34a-5p, miR-181a-5p, and miR-218-5p were downregulated in osteoporotic patients. Importantly, several associations (miR-92a-3p, miR-181a-5p, miR-218-5p) emerged only after multivariable adjustment for clinical parameters, while others (e.g., miR-155-5p) did not remain independent, underscoring that miRNA data cannot be reliably interpreted without rigorous clinical adjustment. Network analysis revealed convergence of deregulated miRNAs on key genes involved in DNA damage response, cell-cycle control, and metabolic stability, including ATM, AURKA, AGO1, AGO4, ALDH9A1, and CUX1. Collectively, these findings support selected miRNAs and their target genes as candidate biomarkers and mechanistic mediators of bone aging and osteoporotic fragility.

## Figures and Tables

**Table 1:** Baseline characteristics.

*Characteristic*	OP Group (Mean ± SD) n=21	Control Group (Mean ± SD) n=25	p-value
*Age (years)*	72 ± 7.29	69 ± 8.06	0.29
*Female*	67 % (n=14)	72 % (n=18)	0.7
*Male*	33 % (n=7)	28 % (n=7)	1
*Smoking (yes, %)* a	40 % (n=10)	43 % (n=9)	0.85
*BMI*	27 ± 3.43	30 ± 4.65	0.01*
*BMD femoral neck (g/cm* *^3^* *)*	1.0 ± 0.18	1.0 ± 0.20	0.56
*CTx ng/l* *b*	938.53 ± 646.8	594.04 ± 285.34	0.04*
*P1NP μg/l* b	93.24 ± 85.51	59.45 ± 24.4	0.07
*25(OH)D (nmol/l)* b	39.4 ± 20.2	44.1 ± 20.6	0.47

a Self-reported information provided by participants.

b Reference ranges of Pardubice Hospital for adults aged 18–99 years: CTX-I 70–700 ng/l; P1NP 20–70 μg/l.

* Statistically significant values (p<0.05).

**Table 2:** Generalized Linear model associations between miRNA expression and clinical parameters.

*Dependent variable*	Predictor	Estimate (β)	SE	*t*-value	p-value
***miR-34a-5p***	Osteoporosis vs. control	2.161	0.635	3.403	**0.001**
	Age (years)	−0.040	0.041	−0.963	0.342
***miR-92a-3p***	Osteoporosis vs. control	0.468	0.215	2.177	**0.038**
	Age (years)	−0.004	0.014	−0.305	0.762
	BMI (kg/m^2^)	−0.030	0.023	−1.299	0.205
	CTX-I (ng/l)	−0.00017	0.00033	−0.509	0.614
	P1NP (μg/l)	−0.00175	0.00271	−0.643	0.525
	BMD (g/cm^2^)	−1.115	0.580	−1.921	0.065
***miR-106a-5p***	Osteoporosis vs. control	1.831	0.859	2.132	**0.041**
	Age (years)	0.047	0.054	0.871	0.391
	CTX-I (ng/l)	−0.00234	0.00134	−1.747	0.091
	P1NP (μg/l)	−0.00180	0.01151	−0.156	0.877
***miR-181a-5p***	Osteoporosis vs. control	−1.282	0.628	−2.042	**0.050**
	Age (years)	−0.015	0.037	−0.404	0.689
	CTX-I (ng/l)	−0.00196	0.00077	−2.535	**0.017**
	BMI (kg/m^2^)	−0.047	0.066	−0.701	0.489
***miR-218-5p***	Osteoporosis vs. control	−1.517	0.689	−2.201	**0.036**
	Age (years)	−0.041	0.041	−1.013	0.319
	BMI (kg/m^2^)	−0.043	0.073	−0.584	0.564
	CTX-I (ng/l)	−0.00125	0.00085	−1.478	0.150

* p<0.05.

** p<0.01.

The estimate refers to the estimated value of each coefficient for the predictors (independent variables) in the model. These estimates represent the relationship between each predictor and the response (dependent variable). Std. Error is a measure of the variability or precision of the estimated coefficients for each predictor variable. The *t*-value is the test statistic for each predictor coefficient. It helps determine whether a given predictor variable significantly contributes to explaining the variance in the response variable. Pr(>|*t*|) represents the p-value associated with the *t*-statistic for a particular coefficient in the model. This p-value indicates the probability that the observed coefficient differs from zero purely by chance.
